# New-Generation Antibacterial Agent—Cellulose-Binding Thermostable TP84_Endolysin

**DOI:** 10.3390/ijms252313111

**Published:** 2024-12-06

**Authors:** Małgorzata Ponikowska, Joanna Żebrowska, Piotr M. Skowron

**Affiliations:** 1 Department of Molecular Biotechnology, Faculty of Chemistry, University of Gdansk, 80-309 Gdansk, Poland; malgorzata.ponikowska@gumed.edu.pl; 2 Department of Biology and Medical Genetics, Faculty of Medicine, Medical University of Gdansk, 80-211 Gdansk, Poland

**Keywords:** thermophage, bacteriophage, TP-84, endolysin, cellulose-binding domain, antimicrobial

## Abstract

The increasing antibiotic resistance among bacteria challenges the biotech industry to search for new antibacterial molecules. Endolysin TP84_28 is a thermostable, lytic enzyme, encoded by the bacteriophage (phage) TP-84, and it effectively digests host bacteria cell wall. Biofilms, together with antibiotic resistance, are major problems in clinical medicine and industry. The challenge is to keep antibacterial molecules at the site of desired action, as their diffusion leads to a loss of efficacy. The TP84_28 endolysin gene was cloned into an expression-fusion vector, forming a fusion gene *cbd_tp84_28_his* with a cellulose-binding domain from the cellulase enzyme. The Cellulose-Binding Thermostable TP84_Endolysin (CBD_TP84_28_His) fusion protein was biosynthesized in *Escherichia coli* and purified. Thermostability and enzymatic activities against various bacterial species were measured by a turbidity reduction assay, a spot assay, and biofilm removal. Cellulose-binding properties were confirmed via interactions with microcellulose and cellulose paper-based immunoblotting. The high affinity of the CBD allows for a high concentration of the fusion enzyme at desired target sites such as cellulose-based wound dressings, artificial heart valves and food packaging. CBD_TP84_28_His exhibits a lytic effect against thermophilic bacteria *Geobacillus stearothemophilus*, *Thermus aquaticus*, *Bacillus stearothermophilus*, and *Geobacillus* ICI and minor effects against mesophilic *Bacillus cereus* and *Bacillus subtilis.* CBD_TP84_28_His retains full activity after preincubation in the temperatures of 30–65 °C and exhibits significant activity up to its melting point at 73 °C. CBD_TP84_28_His effectively reduces biofilms. These findings suggest that integrating CBDs into thermostable endolysins could enable the development of targeted antibacterial recombinant proteins with diverse clinical and industrial applications.

## 1. Introduction

Antibiotic resistance is growing worldwide, and there are at least 2.8 million antimicrobial-resistant infections causing as many as 35,000 deaths yearly in the USA alone [1]. Therefore, innovative approaches employing bacteriophages (phage therapy) and their enzymes to fight bacterial infections are gaining increasing attention. Bacteriophages target specific bacterial strains and dismantle biofilms. They can be used individually, in cocktails, or in combination with other antimicrobials to enhance their effectiveness [2]. Among the diverse mechanisms bacteriophages employ to lyse their hosts, one of the most notable is their ability to bypass the bacterial CRISPR/Cas defence system. This is achieved by deactivating it through the use of anti-CRISPR genes—a discovery with the potential to revolutionize the treatment of antibiotic-resistant infections [3]. There is mounting evidence for the successful use of phages in patients with severe infections that are unresponsive to standard treatment [4,5,6,7,8], and there are many ongoing clinical trials [9]. At present, in most Western countries, phage therapy is only available for emergency or compassionate use. However, legislation is evolving, with Belgium pioneering new regulations to allow phage therapy as a magistral preparation [8,9]. In contrast, countries like Georgia and Russia have made phage cocktails into pharmaceuticals that are available commercially and without prescription [9]. Bacteriophage therapy holds significant potential as a targeted, customizable approach to treat bacterial infections. However, several challenges remain: the complex manufacturing process, phages’ narrow host range, their tendency to mutate, the build-up of bacterial resistance, and the neutralization of highly immunogenic phage nucleoprotein particles by the human immune system [2]. One approach to addressing the problem of the natural biological variability of phages is to isolate their active bacteriolytic enzymes and use these directly to combat bacteria.

Endolysins are bacteriophage-produced lytic proteins, vital for exiting the host cell. Many endolysins have been tested in vitro, providing promising results. To date, none of them has been registered as a drug, but clinical research on the safety and efficacy of endolysins is ongoing [10]. One of the endolysins, *Staphefekt*™, is available in *Gladskin* cosmetics and is registered as a medical device [11]. Endolysins are promising candidates for replacing or complementing traditional antibiotics. Most endolysins target highly conserved bonds within peptidoglycan, making it very difficult for bacteria to evolve endolysin resistance. Exposing bacteria to low endolysin concentrations shows that endolysin resistance development is a very rare event [12,13].

Due to the significant variation in the chemical structure of peptidoglycan in Gram-positive bacteria, the presence of peptidase catalytic and binding domains in Gram-positive specific endolysins often leads to a relatively narrow host range. In contrast, endolysins from Gram-negative-specific bacteriophages are small globular proteins usually lacking a binding domain [14]. They have a broader activity range, which is consistent with the relatively conserved structure of peptidoglycan across Gram-negative bacteria [15].

Four types of bacterial cell wall lytic enzymes can be distinguished, and based on the different types of peptidoglycans, they target the following:

(i) Glycosidases, cleaving the bonds between N-acetylglucosamine and N-acetylmuramic acid, including two subgroups: N-acetylmuramidases and N-acetylglucosaminidases.
(ii) Transglucosylases, cleaving bonds between N-acetylmuramic acid and N-acetylglucosamine (like N-acetylmuramidases), but in a different mechanism; they do not require water and thus are not considered hydrolases.
(iii) Amidases, cleaving the amide bond between N-acetylmuramic acid and L-alanine, the first amino acid in the cross-linking peptide.
(iv) Endopeptidases, cleaving peptide bonds between amino acids [16,17,18,19].

Bacteriophage TP-84 was isolated in 1952 from greenhouse soil, using *Bacillus (B.) stearothermophilus* strain 2184 as the host [20]. This bacterium was recently reclassified as *Geobacillus (G.) stearothermophilus*. The closely related *G. stearothermophilus* strain 10 was further used as a host, since TP-84 exhibits a narrow host range, limited to several *B. stearothermophilus* and *G. stearothermophilus* strains [21,22]. We sequenced its genome, and we proposed to rename it *G. thermoleovorans* strain 10 [23]. However, before the publication of the full genomic sequence, for clarity, we use here the name *G. stearothermophilus* strain 10.

Bacteriophage TP-84 exhibits an extraordinarily wide temperature growth range of 30–80 °C, covering both the mesophilic and thermophilic range, with the optimum at 55–65 °C. TP-84 has the capability to proliferate within the entire temperature range, although with widely varying efficiency [24,25]. We have sequenced the TP-84 genome and bioinformatically characterized its 81 ORFs, 73 of which were confirmed by proteomics analysis [24,25]. Three of those, TP84_26 glycosylase-depolymerase, TP84_27 holin and TP84_28 endolysin, were assigned as hypothetical lytic proteins. TP-84 belongs to the family *Siphoviridae*, and we have shown that it is a genomic orphan with very little sequence similarity to any bacterial or viral genomes at the time of analysis in 2018 [24]. This makes TP-84 an interesting source of novel proteins, which may exhibit properties not observed in those derived from other species [19]. A bioinformatic analysis of the *tp84_28* gene has revealed an enzymatic catalytic domain, GH25_Lyc-like (21–206 aa) [cd06525—protein BLAST alignment on-line 16.10.2024]. Furthermore, there are three LysM superfamily domains [cl21525—protein BLAST alignment on-line 16.10.2024] responsible for peptidoglycan binding, which have also been seen in proteins exhibiting functions other than lysis [19].

We have cloned and expressed the *tp84_28* gene and determined that TP84_28 is a thermostable protein with a melting point of 77.6 °C, which coincides with its loss of lytic activity. The protein is active in a wide range of pH values, from 4.0 to 10.0, with the maximum at 7.5–8.0. In this work, we constructed and evaluated a bioactive fusion protein—CBD_TP84_28_His—consisting of TP84_28 endolysin and a cellulose-binding domain (CBD). CBDs in general are substantially autonomous domains of cellulases, still capable of binding to cellulose if separated from cellulase’s catalytic domains [26]. This feature is retained in a number of fusion protein constructs, where the cellulase catalytic domain is replaced by another protein.

## 2. Results

### 2.1. Cloning, Overproduction, and Purification of CBD_TP84_28_His

**Figure 1:**
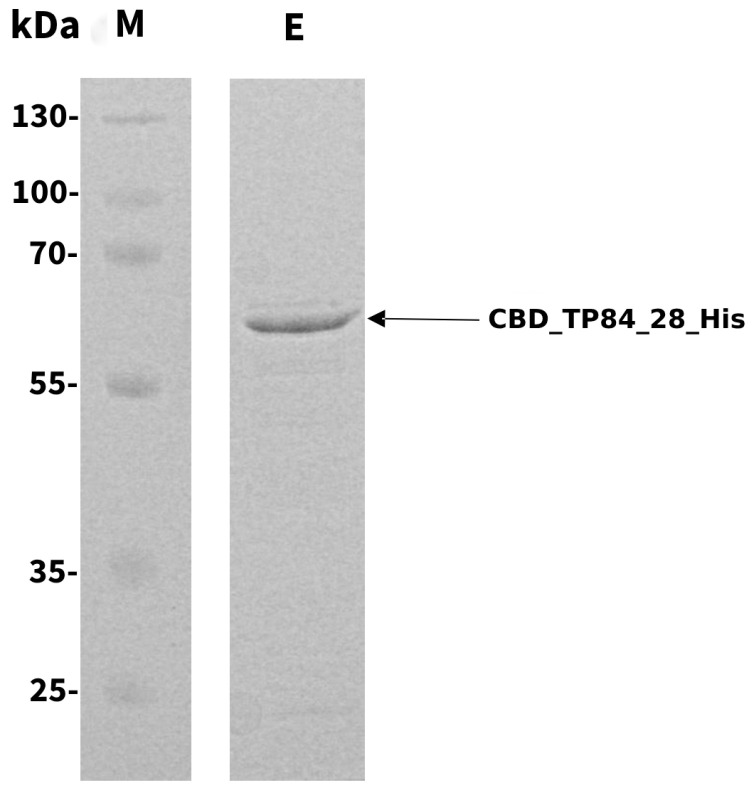
SDS-PAGE analysis of the purified CBD_TP84_28_His. M, PageRuler Plus Stained Protein Ladder; E, CBD_TP84_28_His. Arrow points at a band corresponding in size to the endolysin CBD_TP84_28_His (64.2 kDa).

The pET28_del*Sap*I_CBD_His (Supplementary Figure S1) expression clones coding for CBD_TP84_28_His were stably maintained in various *Escherichia (E.) coli* strains. However, IPTG-induced cultures of *E. coli* BL21 (DE3) pET28_CBD_TP84_28_His exhibited slower growth rate compared to both uninduced culture and control *E. coli* BL21 (DE3) culture, devoid of the pET28_CBD_TP84_28_His plasmid (Supplementary Figure S2). This indicates moderate toxicity to the *E. coli* host, similar to what we have observed during the expression of recombinant, but not-fused TP84_28_His endolysin [19]. The simple purification protocol, which included the removal of nucleic acids and acidic protein by polyethyleneimine (PEI) precipitation, followed by metal affinity chromatography on an NiNTA HisTrap HP column, enabled us to obtain nearly homogeneous CBD_TP84_28_His (over 95%) (Figure 1). The purified preparation was stored at 4 ℃ for more than 3 months and at −80 ℃ with added glycerol (10%) for over 6 months, without the loss of activity. The final yield was 2 mg (concentration 0.5 mg/mL) of an enzymatically active recombinant protein from 1 L of the expression culture.

### 2.2. Properties of CBD_TP84_28_His

#### 2.2.1. Lytic Activity of the Recombinant Fusion Endolysin CBD_TP84_28_His Against Test Bacteria

**Figure 2:**
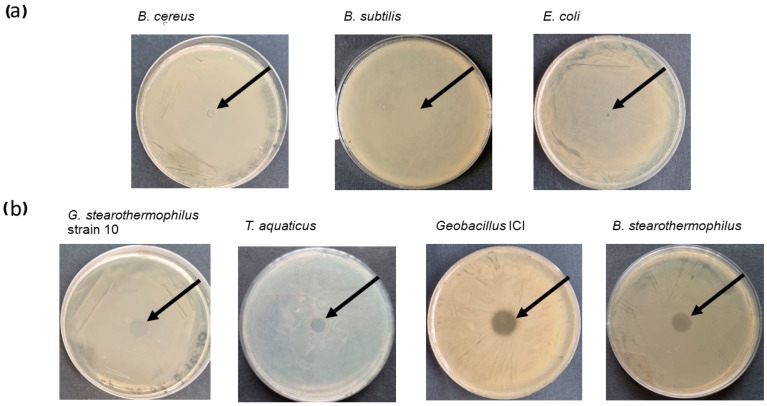
Recombinant fusion endolysin CBD_TP84_28_His activity evaluation—spot assay. Top row (**a**) shows activity of CBD_TP84_28_His against mesophilic bacteria: *B. cereus*, *B. subtilis*, *E. coli.* Bottom row (**b**) shows activity of CBD_TP_84_28_His against thermophilic bacteria: *G. stearothermophilus* strain 10, *T. aquaticus*, *Geobacillus* ICI, *B. stearothermophilus.* Arrows indicate where CBD_TP84_28_His solution was spotted and transparent circles on the bacterial lawn indicate lytic effect of the enzyme.

**Figure 3:**
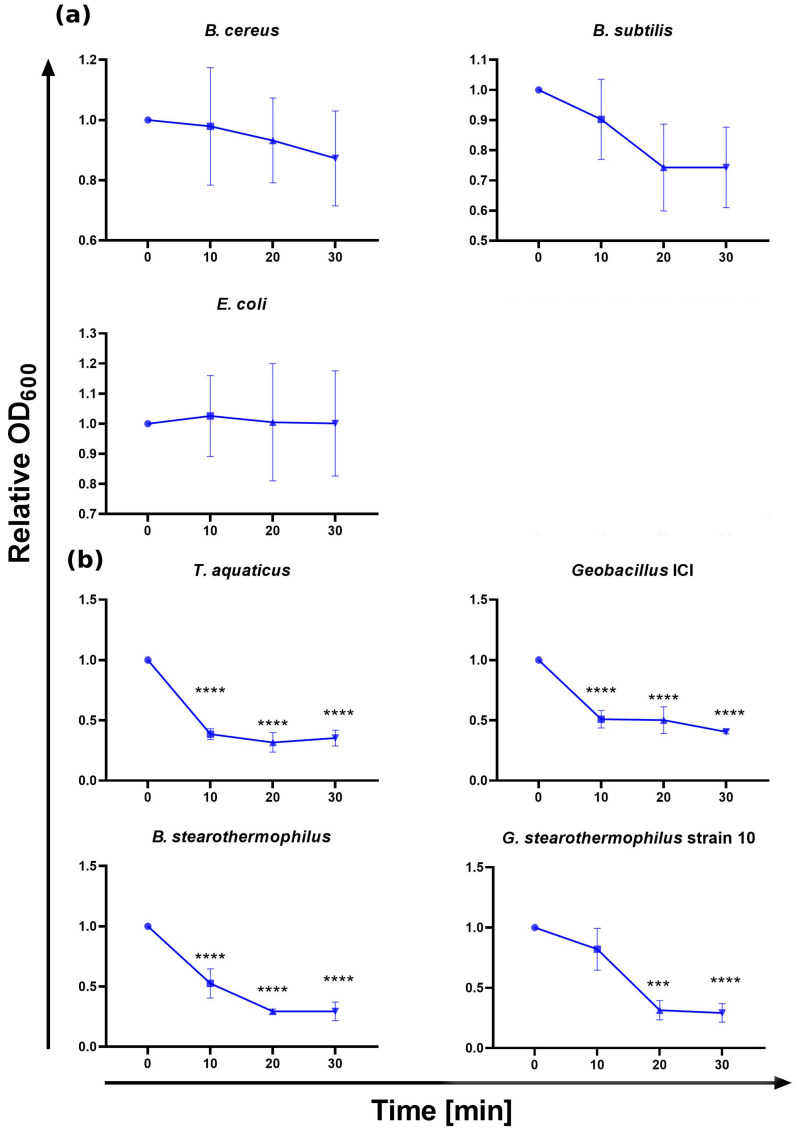
CBD_TP84_28_His activity evaluation—TRA on various bacterial strains. The graph shows the reduction in relative OD_600_ (ratio of OD_600_ of the sample treated with CBD_TP84_28_His to the OD_600_ of the control) in bacterial substrate suspensions upon addition of purified CBD_TP84_28_His to the final concentration of 1.43 µg/mL at 55 °C. Top row (**a**) shows activity of CBD_TP84_28_His against mesophilic bacteria: *B. cereus*, *B. subtilis*, *E. coli*. Bottom rows (**b**) show activity of CBD_TP_84_28_His against thermophilic bacteria: *G. stearothermophilus* strain 10, *T. aquaticus*, *Geobacillus* ICI, *B. stearothermophilus*. Results are presented as means ± SD of three independent experiments. Statistically significant differences were determined by one-way ANOVA with Dunnett’s test and marked as (***)—*p*-value  < 0.001, (****)—*p*-value  < 0.0001.

Two methods of testing lytic activity of CBD_TP84_28_His against bacteria were used: the qualitative spot assay (diffusion test) and quantitative turbidity reduction assay (TRA). The spot assay results obtained for bacteria incubated at 42 °C showed no lytic effect of CBD_TP84_28_His against *B. subtilis.* A minor lytic effect was observed against *E. coli* and a moderate lytic effect was observed in the case of *B. cereus.* A considerable lytic effect was visible for *G. stearothermophilus* strain 10, *Thermus (T.) aquaticus, Geobacillus* ICI, and *B. stearothermophilus* (Figure 2). To exclude the influence of incubation temperature on the activity of CBD_TP84_28_His, its lytic potency was also assessed via the TRA. After centrifugation and resuspension in buffer R, each tested bacterial strain was subjected to digestion by CBD_TP84_28_His at 55 °C (Figure 3). A reduction in resuspended bacteria OD_600_ upon the addition of CBD_TP84_28_His progressed rapidly for *T. aquaticus*, *B. stearothermophilus*, *Geobacillus* ICI and *G. stearothermophilus* strain 10 (Figure 3). Though a minor lytic effect was observed via a visual inspection of the fresh plates in the case of *B. cereus* and *B. subtilis*, it did not reach statistical significance in the TRA (Figure 3), just as in the case of *E. coli,* where the relative test culture OD_600_ was stable across measurements.

#### 2.2.2. Comparison of the Activity of the CBD_TP84_28_His with the Recombinant Endolysin TP84_28_His

**Figure 4:**
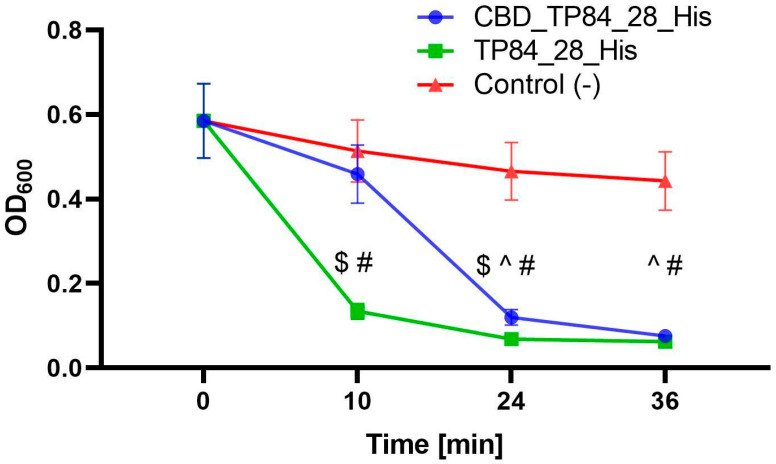
Comparison of activity of the CBD_TP84_28_His and recombinant TP84_28_His. The graph shows reduction in OD_600_ in *G. stearothermophilus* strain 10, resuspended in buffer R, after addition of equimolar amounts of CBD_TP84_28_His, TP84_28_His or the reaction buffer alone [control (-)]. Results are presented as means ± SD of three independent experiments. Statistical significance was assessed using two-way ANOVA with Tukey’s post hoc test, with symbols indicating significant differences (*p*-value < 0.05) between groups: ($)—CBD_TP84_28_His vs. TP84_28_His, (^)—CBD_TP84_28_His vs. control (-), (#)—TP84_28_His vs. control (-).

To evaluate the effect of fusing the CBD to endolysin TP84_28_His on lytic activity, equimolar amounts of CBD_TP84_28_His and recombinant TP84_28_His were used to perform the TRA. The results indicated that the endolysin TP84_28_His digests *G. stearothermophilus* strain 10 2–3 times faster than the bimodular CBD_TP84_28_His in the linear activity range between 5 and 10 min (Figure 4).

#### 2.2.3. Lytic Activity of the CBD_TP84_28_His Against Biofilm

- The Inhibition of biofilm formation by *G. stearothermophilus* strain 10

**Figure 5:**
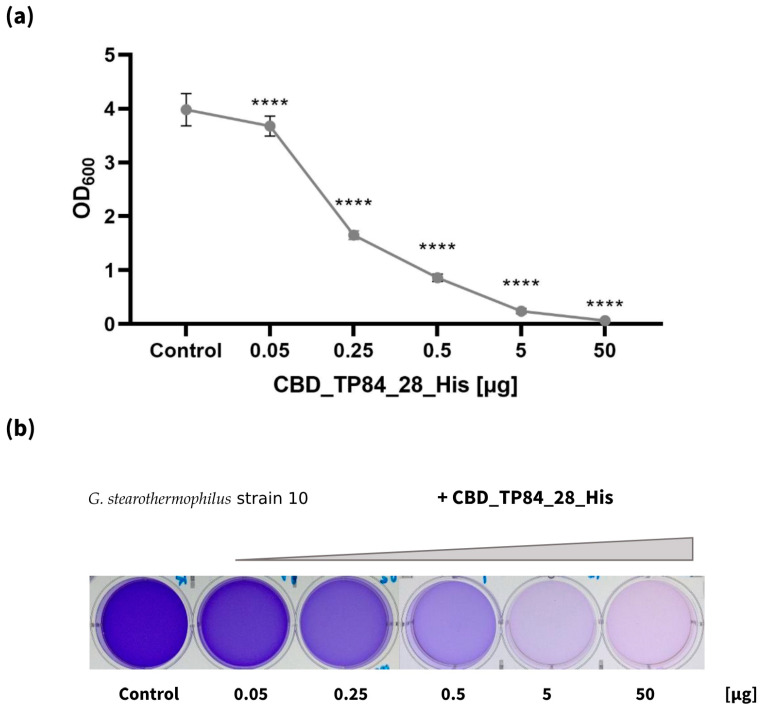
Inhibition of biofilm formation by *G. stearothermophilus* strain 10. The graph (**a**) shows OD_600_ measurements of *G. stearothermophilus* strain 10, cultivated on microtiter plate with addition of 0.05–50 µg of CBD_TP84_28_His for 24 h at 55 °C. The picture below the chart (**b**) shows biofilm stained with crystal violet: control on the left and samples treated with increasing amounts of the recombinant fusion endolysin CBD_TP84_28_His. Results are presented as means ± SD of three independent experiments. Statistically significant differences were determined by one-way ANOVA with Dunnett’s test and marked as (****)—*p*-value < 0.0001.

This assay was conducted in the microtiter plate format for the quantitative determination of required CBD_TP84_28_His amounts for efficient biofilm digestion. This was achieved by adding various amounts of CBD_TP84_28_His to biofilms formed in a microtiter plate. In total, 0.05 µg of CBD_TP84_28_His reduced biofilm formation by *G. stearothermophilus* strain 10 by 10%, and adding 0.25 µg reduced it by 59%, 0.5 µg by 79% and 5 µg by 96% and 50 µg by a 98% reduction in biofilm formation, respectively (Figure 5).

- The Inhibition of biofilm formation by mesophilic pathogenic-related bacteria

The effect of CBD_TP84_28_His on the formation of mesophilic bacterial biofilms was investigated. For safety reasons, these were formed from bacterial strains closely related to real pathogenic bacteria. CBD_TP84_28_His showed the greatest inhibition of biofilm formation by *Staphylococcus (S.) aureus*, but this much lower for *Pseudomonas (P.) aeruginosa* and *E. coli*. However, for *Salmonella (S.) enteritidis* and *B. cereus*, the inhibition of biofilm formation was slightly above 30% (Table 1).

#### 2.2.4. Thermostability of the CBD_TP84_28_His

**Figure 6:**
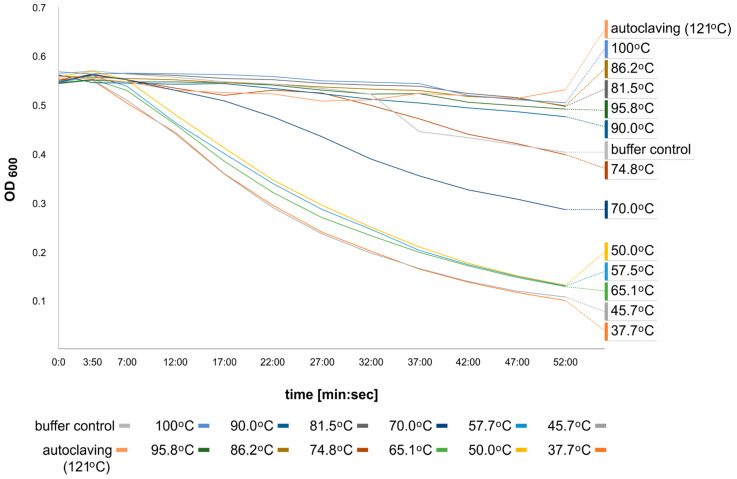
CBD_TP84_28_His thermal stability evaluation. The lytic activity of CBD_TP84_28_His was assayed in optimal conditions after preincubation at various temperatures in a gradient thermocycler. The graph shows a reduction in OD_600_ in *G. stearothermophilus* strain 10 suspension in buffer R after the addition of CBD_TP84_28_His, preincubated for 30 min at temperatures of 37.7–95.8 °C and autoclaving conditions (121 °C, 20 min). As a negative control, buffer R was added instead of CBD_TP84_28_His. Measurements were taken using the Tecan microplate reader. This graph shows the final single-repeat experiment, including all the temperature points. Prior to the combined experiment, each temperature was evaluated with 3 repetitions separately. This arrangement was necessary to eliminate uncontrolled reaction progression due to the high number of samples (14) needing to be processed simultaneously.

The thermostability of CBD_TP84_28_His was evaluated at 12 temperature values in the range of 30–100 °C, autoclaving for 30 min in buffer R (50 mM Na/PO_4_, 150 mM NaCl, pH = 7.0), followed by the TRA. Similar rates of reaction were observed for samples preincubated in the temperature range 30–65.1 °C. A significant reduction in the reaction rate was noted for the sample preincubated at 70 °C, and a further reduction in the reaction rate was observed for the sample incubated in the temperature of 74.8 °C (Figure 6). A further increase in the preincubation temperature caused very similar results as for 74.8 °C. The observed reduction in the activity aligns with the thermostability of the studied protein: the protein starts to unfold at 64.98 °C, with a melting point of 73.06 °C. We determined these temperatures using an alternative method: nano differential scanning fluorimetry (nano-DSF) using a Prometheus Panta device (NanoTemper, Munich, Germany).

#### 2.2.5. Cellulose-Binding Properties of CBD_TP84_28

- Interaction with microcellulose (µC) assay
- Cellulose paper-based immunoblotting

## 3. Discussion

The functionalization of the thermostable TP84_28 with a CBD for antimicrobial applications, such as cellulose-based wound dressings, offers distinct advantages over alternatives like mesophilic T4 lysozyme fused with a CBD [27]. The TP84_28 endolysin, derived from the bacteriophage TP-84, exhibits high thermostability, up to 77.6 °C. This makes it suitable for applications in environments that involve high heat exposure, deactivating mesophilic enzymes [19]. Recombinant CBD_TP84_28_His was expressed in the T7 promoter-based expression system. The yield of 2 mg/1 L culture was rather low due to the expected and apparent toxicity of the endolysin part of the fusion protein against *E. coli*. The biosynthesis of the fusion endolysin slowed down bacterial growth by 35%. In contrast, when the enzyme was applied to *E. coli* from outside in the diffusion assay, it was not able to effectively lyse *E. coli* (Figure 2 and Figure 3). Apparently, CBD_TP84_28_His can penetrate the cytoplasmic membrane of the *E. coli* cell and digest peptidoglycan from inside, whereas the outer membrane forms a non-permeable barrier for the enzyme. There are profound differences between endolysins derived from bacteriophages targeting Gram-positive and Gram-negative bacteria due to the architecture of the cell wall [14]. It was also observed that *P. aeruginosa*, a Gram-negative species, was not susceptible to CBD_TP84_28_His. This is in line with the finding that CBD_TP84_28_His exhibits the highest lytic activity towards thermophilic Gram-positive bacteria related to the host of TP-84 bacteriophage, *G. stearothermophilus* strain 10, *Geobacillus* ICI and *B. stearothermophilus*, similar to unfused TP84_28_His endolysin [19].

*T. aquaticus,* even though not related to *Geobacillus,* is also highly sensitive to CBD_TP84_28_His as it is Gram-positive and therefore without an external membrane (Figure 2 and Figure 3). However, the fact that activity against *T. aquaticus* was comparable to activity against the reference species *G. stearothermophilus* strain 10 points to an interesting, putative difference in enzymatic specificity when compared to unfused TP84_28_His, for which activity against *T. aquaticus* was moderate [19]. A possible reason for this finding may lie in the way that the presence of the CBD affects the folding of the endolysin moiety itself. Slight changes in conformation might shift catalytic preference to other peptidoglycan structural variants. The ability of CBD_TP84_28_His to target Gram-positive bacteria, including antibiotic-resistant and biofilm-forming strains, is particularly advantageous in clinical settings, where biofilm-associated infections are a challenge. This property is further enhanced when coupled with a CBD, as the enzyme can anchor onto cellulose-based wound dressings, providing localized and sustained antibacterial action. Prolonged retention at the wound site ensures continuous antimicrobial activity.

The activity of CBD_TP84_28_His against mesophilic Gram-positive bacteria related to *G. stearothermophilus* strain 10 remains low and statistically insignificant. In the diffusion test, this may be attributed to the conditions favouring cell wall regeneration/bacterial regrowth (bacterial medium availability and a temperature of 42 °C) rather than lysis by the applied CBD_TP84_28_His, which has a higher optimum temperature range. In the TRA, however, to slow down the regeneration process, the buffer optimal for the enzyme CBD_TP84_28_His was used instead of media. Additionally, the temperature was optimal for the enzyme (55 °C), not for the bacteria, yet the enzyme’s activity against the mesophiles remained low.

The poor activity of CBD_TP84_28_His against mesophilic *Bacillus* spp. Is somewhat surprising, as these are Gram-positive bacteria related to *G. stearothermophilus* strain 10. This points to the following conclusions:

(i) The specificity of CBD_TP84_28_His appears to depend both on the thermophilicity and phylogenetic relatedness of the bacteria.
(ii) The structure of peptidoglycan varies substantially across *Bacillus* bacterial species.
(iii) The structure of peptidoglycan of the tested thermophiles has common features, sensitive to CBD_TP84_28_His.
(iv) Differences in external polysaccharide envelopes may play an important role, preventing the enzyme’s access to the cell wall.

Given the limited activity of CBD_TP84_28_His against mesophilic bacteria, a CBD-fused T4 bacteriophage lysozyme could serve as a complementary or alternative enzyme [27] to be mixed with CBD_TP84_28_His.

The lytic activity of CBD_TP84_28_His against two common pathogenic-related strains was tested and not detected (Gram-negative *P. aeruginosa* and Gram-positive *S. aureus*; this is consistent with the conclusions above.

Endolysins derived from Gram-positive-targeting bacteriophages can be artificially enhanced in accessing the cell wall of Gram-negative species in multiple ways. Pretreatments with high hydrostatic pressure, EDTA, or organic acids all weaken the outer membrane of Gram-negative bacteria [28,29], but these methods are not applicable in clinical settings. More promising approaches include combining endolysins with antibiotics for a synergistic effect; packing endolysins into liposomes to help them cross the outer membrane; or combining them with silver nanoparticles to disrupt the outer membrane [28,29]. Modular proteins fused with an additional domain disrupting the outer membrane have also been reported [12,30].

*Artilysins^®^*, a concept by Briers et al. [31], are endolysins with an additional polycationic nanopeptide dedicated to destabilization of LPSs, a component of the outer membrane. *Artilysins^®^* are an antimicrobial technology platform and Lysando, the biotechnology company that develops the concept, claims that “it is possible to design specific *Artilysin^®^* to target nearly every bacterial species” [32].

Innolysins are endolysins fused with proteins binding to the receptors in the outer membrane of Gram-negative bacteria and are reported to have lytic activity against Gram-negative *P. aeruginosa* [33]. It seems that expanding the functionality of endolysins by adding new moieties and creating a fusion protein is one way to pursue the desired effect of the potential drug.

Although CBD_TP84_28_His has not shown high activity against the tested pathogen-related bacteria responsible for nosocomial infections, it has shown lytic activity towards the biofilm formed by all the bacteria tested, including highly pathogenic *S. aureus* (Table 1). A biofilm is a mixture of microbial cells, exopolysaccharides, DNA and proteins, which serves as a protective milieu for the bacteria and so poses a major clinical problem. Somewhat surprisingly, the highest reduction in biofilm was observed for the *S. aureus* (81%) and secondly for *P. aeruginosa* (42%)*—*both of which were strains not sensitive to CBD_TP84_28_His in the diffusion test and TRA. The main component of the *S. aureus* biofilm and the only exopolysaccharide is polysaccharide intercellular adhesin (PIA, PNAG)—a partially deacetylated polymer of β-1,6-N-acetylglucosamine [34], suspected to be the target for CBD_TP84_28_His. PIA is also known to be produced by species such as *E. coli, Acinetobacter baumanii, Yersinia pestis,* and *Bordatella* spp. [35]. Therefore, CBD_TP84_28_His could be expected to demonstrate lytic activity against these species, but this requires further testing. The biofilm of *P. aeruginosa* is known to consist of several types of exopolysaccharides [34].

Biofilm formation is a serious problem associated with the long-term use of various medical devices and implants. A biofilm forms a barrier that can be impossible to lyse, even with the application of broad-spectrum antibiotics [36]. Additionally, due to the effort to reduce the plastic burden, immunologically neutral cellulose-based materials are going to become increasingly popular in the construction of such equipment. 

**Figure 7:**
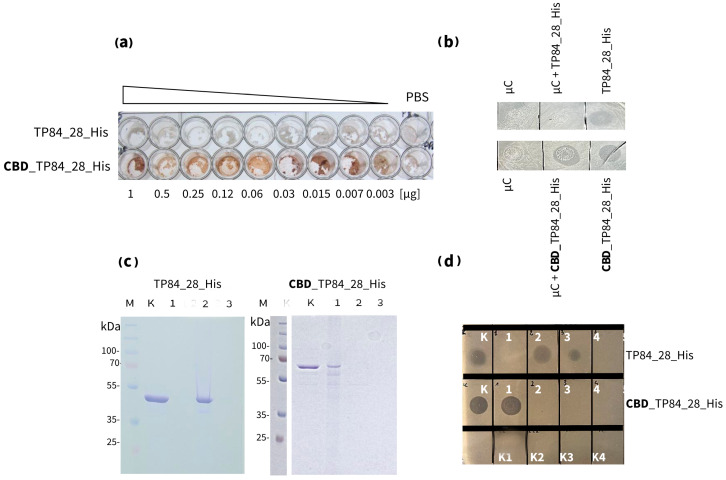
Interactions comparison of recombinant enzymes: endolysin TP84_28_His and CBD_TP84_28_His with µC. (**a**) The binding of TP84_28_His and CBD_TP84_28_His to µC in a 96-well plate using the detection of His-tag for Western blots (Method 2.2.3.1). Both proteins have a His-tag that is located at the C-terminus, which allows for the detection of the protein attached to the µC using anti-His antibodies. (**b**) A dot blot assay of endolysin activity using the host *G. stearothermophilus* strain 10. A control—µC and 1 × PBS dot—was applied, and µC complexes formed with each of the enzymes TP84_28_His and CBD_TP84_28_His, which were applied to the agar plate with spread *G. stearothermophilus* strain 10. (**c**) An SDS-PAGE comparative analysis of the formation of insoluble µC complexes with CBD_TP84_28 and with TP84_28_His. Lane M, PageRuler Plus Stained Protein Ladder; lane K, untreated TP84_28_His (**left**)/CBD_TP84_28 (**right**); lane 1, insoluble µC complex formed with TP84_28_His (**left**)/CBD_TP84_28_His (**right**); lane 2, supernatants of complex formation—unbound protein; lane 3, washing the supernatant of the complexes with 1 × PBS. (**d**) µC complex formed with each of the enzymes, TP84_28_His and CBD_TP84_28_His, which were applied to the agar plate with spread *G. stearothermophilus* strain 10 to measure µC complex activity; dot K, TP84_28_His/CBD_TP84_28_His; dot 1, formed and washed µC complexes with TP84_28_His/CBD_TP84_28_His; dot 2, supernatants of insoluble µC complex formation; dot 3, washes of the complexes with 1 × PBS; dots 4, first washes of the complex with water; K1, control of buffer R; K2, control of µC; K3, control of PBS; K4, control of buffer A.

An example of a potential advanced application of CBD_TP84_28_His is treating infectious endocarditis: this life-threatening condition is often not susceptible to antibiotics due to biofilm formation on artificial heart valves. If innovative cellulose-based medical devices and implants, such as the artificial heart valves just patented [37], become popular, CBD_TP84_28_His has a high potential to become an effective treatment for associated infections. Cellulose would serve as a scaffold for CBD_TP84_28_His, keeping the antibacterial activity highly localized and therefore highly efficient (Figure 7). This approach minimizes potential interactions with off-target tissues and limits the enzyme’s degradation before reaching the target site. The biofilm-disrupting properties of CBD_TP84_28_His could also facilitate the efficacious application of life-saving antibiotics.

**Figure 8:**
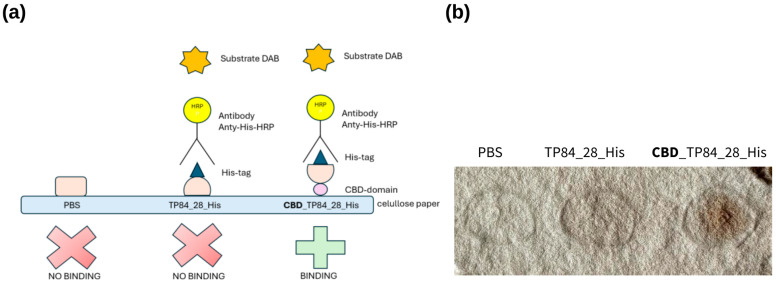
CBD_TP84_28_His interaction with cellulose paper: (**a**) scheme showing CBD binding to cellulose filter paper and His-Tag detection with anti-His-HRP antibody, which enables visualization of the reaction after colour development using 3,3′-diaminobenzidine (DAB). (**b**) Cellulose filter paper with spots: PBS buffer (control), TP84_28_His and CBD_TP84_28_His.

There are also promising prospective applications for CBD_TP84_28_His in food production: the industry will inevitably be switching to alternatives to plastic for packaging solutions, many of which are cellulose derivatives (Figure 8). Target genera of the fusion endolysin, *Bacillus* and *Geobacillus* are responsible for the spoilage of various types of food [38,39]. The CBD_TP84_28_His preserves its full activity in temperatures as high as 65 °C (Figure 6). These results are confirmed by qualitative spot tests (Supplementary Figure S3), which also prove that some residual CBD_TP84_28_His activity is preserved even after autoclaving (121 °C). Measurements using nano-DSF technology confirmed that the melting point of the protein is 73.06 °C, which is slightly less than that of lone TP84_28_His at 77.6 °C [19]. The decrease in the melting point of the fusion protein is most likely due to the presence of the additional cellulose-binding domain. Thermostability gives CBD_TP84_28_His a further advantage as it can be stored or used without a decrease in activity in variable conditions. This feature has been of scientific interest recently, and reports on other thermostable endolysins have been released: LysAP45 [40], LysCPQ7 [41]. Thus, our future research will include comparative studies using various thermostable endolysins, including those coded by novel thermophilic bacteriophages found by our group. 

## 4. Materials and Methods

### 4.1. Bacterial Strains, Media, Reagents, DNA, Software and Devices

Bacteriophage TP-84 (NC_041918.2), its host, *G. stearothermophilus* strain 10 (BGSC No. 9A21, NCBI ID 272567), and bacteria used to assess CBD_TP84_28_His lytic activity came originally from Epstein and Campbell, 1975. *E. coli* BL21 (DE3) (F^−^ ompT hsdS (r_B_-m_B_^−^) dcm^+^ Tet^r^ galλ (DE3) endA Hte) (Agilent Technologies, CA, USA) was used for gene expression, and *E. coli* DH5α (F-φ80*lac*ZΔM15 Δ (*lac*ZYA*-arg*F) U169 *deo*R *rec*A1 *end*A1 *hsd*R17 (r_k_^−^, m_k_^+^) phoA supE44 λ- thi-1 *gyr*A96 *rel*A1 Mrr-) from New England Biolabs (Ipswich, MA, USA) was used for plasmid propagation. For the CBD_TP84_28_His lytic activity measurement, we used the following bacteria: *E. coli* (KPD 168-BA, Gdansk, Poland), *B. stearothermophilus* (KPD 109-BA, Gdansk, Poland)*, B. cereus* (KPD 110-BA, Gdansk, Poland)*, B. subtilis* (KPD 112-BA, Gdansk, Poland) and *Geobacillus* ICI (KPD 1699, Gdansk, Poland). *T. aquaticus* DSM 625 was obtained from the DSM collection (Brunswick, Germany). For culturing *G. stearothermophilus* strain 10, *B. cereus*, *B. subtilis*, *E. coli*, and *B. stearothermophilus,* LB medium (1% tryptone, 0.5% yeast extract, 1% NaCl (BTL, Lodz, Poland)) was used, for *T. aquaticus,* TM medium (0.3% tryptone, 0.2% yeast extract, 1× Castenholz salts(BTL, Lodz, Poland)) was used, for *Geobacillus* ICI, 2×YT medium (1.6% tryptone, 1% yeast extract, 0.5% NaCl (BTL, Lodz, Poland)) was used, and their corresponding solid media were supplemented with 1.5% of agar. For the bacterial biofilm disruption assessment, TSBg medium (1.7% peptone K; 0.3% peptone SP; 0.5% NaCl; 0.25% K_2_HPO_4_; 0.25% glucose, pH 7.3 (BTL, Lodz, Poland)) was used. Bacterial strains used to assess the activity of fusion protein CBD_TP84_28_His against biofilm included the host strain *G. stearothermophilus* strain 10 and bacteria related to those known for their pathogenicity for humans: *E. coli* DSM 1103 and *B. cereus* DSM 31 from DSM collection, as well as *S. aureus* ATCC 25923, *P. aeruginosa* ATCC 17503, *S. enteritidis* ATCC 25928 from the ATCC collection (Manassas, VA, USA). The Plasmid Mini kit and Gel-Out ACX kit were from A&A Biotechnology (Gdansk, Poland). *Sap*I restriction endonuclease, alkaline phosphatase, T4 ligase, Q5^®^ High Fidelity DNA Polymerase and Q5^®^ High GC Enhancer and dNTPs were from New England Biolabs (Ipswich, MA, USA). PCR primer synthesis, DNA cassete synthesis and DNA sequencing were conducted at Eurofins Genomics (Ebersberg, Germany). T4 DNA Ligase and low-melting-point agarose were supplied by ThermoFisher Scientific Baltics UAB (Vilnus, Lithuania). Agarose was from Bioshop (Burlington, ON, Canada). VivaSpin Turbo membranes came from Sartorius (Burlington, NJ, USA). Chromatographic column Ni-NTA HisTrap HP was obtained from GE Healthcare Bio-Sciences AB (Uppsala, Sweden). The Clone ID kit was from Lucigen Corporation (Middleton, WI, USA). Genetic constructs were designed with SnapGene 4.1 software. Microplate reader Infinite M200 PRO by Tecan Trading AG (Männedorf, Switzerland), Victor3 V multilabel plate reader (Waltham, MA, USA) and spectrophotometer Jenway 7205 by Cole-Parmer Instrument Company, LCC (St Neots, UK) were used for spectrophotometric measurements. T100 Thermal Cycler and next-generation chromatography apparatus were supplied by Bio-Rad (Hercules, CA, USA). The Prometheus Panta device by NanoTemper Technologies (Munich, Germany) was used for nano-DSF to measure the unfolding temperature of the fusion CBD_TP84_28_His. Moreover, 6-well, flat-bottom polystyrene microplates by Nest Scientific Biotechnology (Rahway, NJ, USA) were used to pour bacterial solution and then to assess biofilm formation inhibition. Corning^®^ 96-well Clear Flat-Bottom Polystyrene High-Bind Microplate purchased from Corning (Corning, NJ, USA) was used to place µC when assessing its interactions with CBD_TP84_28_His. To visualize interactions of CBD_TP84_28_His with cellulose materials using dedicated antibodies, EveryBlot Blocking Buffer from Bio-Rad (Hercules, CA, USA), DAB from Sigma-Aldrich (Saint Louis, MO, USA) and anti-His HRP-conjugated antibodies from Merck (A7058; RRID-AB_2563555) (Darmstadt, Germany) were used. The µC used to assess the affinity of the fusion CBD_TP84_28_His to cellulose was prepared as we described before [19]. All other chemical reagents, antibiotics, lysozyme were purchased from Merck (Darmstadt, Germany).

### 4.2. Construction, Expression and Purification of Fusion Endolysin—CBD_TP84_28_His

#### 4.2.1. Cloning *tp84_28* Gene into pET28_delSapI_CBD_His Vector

The fusion gene, coding for fusion protein CBD_TP84_28_His, was constructed by cloning using the pET28_del*Sap*I_CBD_His custom-made expression fusion vector (a derivative of the pET21d (+) vector) for cloning the PCR-amplified *tp84_28* gene. The vector contains the sequence coding for the CBD, derived from the cellulase of *Clostridium cellulovorans* [26] in perfect fusion with the vector’s START codon. The enterokinase site was included between sequences coding for the CBD and TP84_28_His domain, and the His6-tag was included at the C-terminus of the recombinant protein. The resulting fusion ORF was under the control of the T7 promoter. The *tp84_28* gene was amplified in a PCR using TP-84 genomic DNA as a template, Q5^®^ High Fidelity DNA Polymerase, Q5^®^ High GC Enhancer and the following primers: F-pET_TP28_*Sap*I_F-gagctcttcacccgggatgcaagcaagat, R-pET_TP28_*Sap*I_R-CATGCTCTTCTGGGCCCgtttggaattttgatcgt. The PCR temperature conditions were denaturing at 98 °C, 30 cycles: 98 °C per 30 s, 62 °C per 30 s, 72 °C per 2 min, and annealing 72 °C per 4 min. The reaction product was phenol–chloroform purified and ethanol precipitated. The vector was *Sap*I digested, dephosphorylated using *E. coli* bacterial alkaline phosphatase, separated along with the *Sap*I-digested PCR product on agarose electrophoresis, both Gel-Out AX kit (A&A Biotechnology, Gdansk, Poland)-purified and ligated using T4 ligase at the insert/vector molar ratio 6:1 at 16 °C for 16 h. The mixture was transformed into *E. coli* DH5α, plated onto kanamycin-supplemented (30 μg/mL) LB plates, and the resulting clone PCRs were analysed using the Clone ID kit with primers pET-tert (TGCTAGTTATTGCTCAGCGG) and pET-up (GATGCGTCCGGCGTAGA). The reaction conditions included the following: 25 PCR cycles, denaturation at 98 °C for 2 min, annealing at 52 °C for 30 s, elongation at 72 °C for 1 min, and finally, fill-in at 72 °C for 3 min. The resulting pET28_del*Sap*I_CBD_TP84_28_His clones were sequenced, and isolated plasmid DNA was transformed into expression strain *E. coli* BL21 (DE3), previously used for the overproduction of TP84_28 endolysin [19]. The sequence of pET28_del*Sap*I_CBD_TP84_28_His was submitted to GeneBank under the accession number PP894717.

#### 4.2.2. Gene Expression and Overproduction of CBD_TP84_28_His 

A 100 mL overnight culture (16 h) of *E. coli* BL21 (DE3) pET28_CBD_TP84_28_His was started from a single bacterial colony in LB medium, supplemented with kanamycin (30 µg/mL). It was used to inoculate 1 L of freshly prepared LB medium with kanamycin (30 µg/mL) to optical density at wavelength 600 nm (OD_600_) 0.1. The culture was grown until OD_600_ = 0.6, induced with 1 mM isopropyl-β-D-1-thiogalactopyranoside (IPTG), and cultivated for 4 h at 30 °C, and cells were harvested by centrifugation (2739× *g*, 15 min, 10 °C).

#### 4.2.3. Recombinant Fusion Endolysin CBD_TP84_28_His Purification

Bacterial cells (1.6 g) were resuspended in buffer A (50 mM Tris-HCl pH = 7.0, 500 mM NaCl) in a mass ratio of 1:10. Phenylmethylsulphonyl fluoride (PMSF) was added to the final concentration of 1 mM and lysozyme to the final concentration of 0.5 mg/mL, and the mixture was incubated for 30 min at 4 °C. Lysis was completed by sonication, with ice-cooling the lysate, which was subsequently centrifuged (16,000× *g*, 20 min, 4 °C) and filtered through a 0.22 µm syringe filter. Further purification of the recombinant CBD_TP84_28_His protein was performed by removing the thermolabile *E. coli* proteins by heating at 55 °C for 10 min and centrifugation (12,000× *g*, 30 min, 10 °C). The bulk of the other proteins contaminating the supernatant and fragmented nucleic acids were precipitated with 0.2% PEI buffered to pH 8.0. Precipitate was removed by centrifugation (centrifugation 12,000× *g*, 30 min, 10 °C) and dialysed to buffer A: 50 mM Tris-HCl, 500 mM NaCl (pH = 7.0). The CBD_TP84_28 was further metal affinity-purified using NGC chromatography and a 5 mL Ni-NTA HisTrap HP column. In total, 15 mL of the solution containing the protein of interest was applied on the column; the column was washed with buffer A (10 column volume (CV)), and CBD_TP84_28_His was eluted with imidazole gradient (20 CV) from 0 mM to 500 mM. The recombinant CBD_TP84_28_His was eluted at 300–400 mM imidazole concentration. Further processing included sample concentration on 10 kDa polystyrene ultrafiltration units while changing buffer R (50 mM Na/PO_4_, 150 mM NaCl, pH = 7.0). SDS-PAGE analysis was performed to assess the purity of the protein. 

### 4.3. Characterization of CBD_TP84_28_His

#### 4.3.1. Evaluation of the Lytic Activity of CBD_TP84_28_His

- Spot assay (diffusion test)

Petri dishes with agar medium for the test bacteria were prepared, (i) LA for *E. coli, B. subtilis, B. cereus, B. stearothermophilus,* and *G. stearothermophillus* strain 10; (ii) 2xYT solid medium for *Geobacillus* ICI; and (iii) TM solid medium for *T. aquaticus,* and 100 µL of fresh bacterial culture was rubbed into the plate. Bacteria were cultivated at their optimal temperatures (*E. coli*, *B. subtilis*, and *B. cereus* at 37 °C, *G. stearothermophillus* strain 10 at 55 °C, and *B. stearothermophilus, T. aquaticus*, and *Geobacillus* ICI at 60 °C) for variable periods of time until they formed a uniform bacterial lawn. After that, 10 µL of PBS (control) or 10 µL of CBD_TP84_28_His preparation was spotted in the middle of the Petri dish and allowed to dry for 40 min. The plates were left for 3 h at the selected temperature for the tested bacteria, and the lytic activity of CBD_TP84_28_His was documented by photographing.

- Turbidity reduction assay (spectrophotometer variant)

A spectrophotometer-quantified TRA was used [19]. Overnight (16 h), cultures of the test bacteria were grown with vigorous aeration at corresponding media and temperatures (Section 4.1 and Section 4.3.1). Then, they were used to inoculate fresh medium of the same type, which was cultivated until the mid-exponential phase (OD_600_ = 0.6). The cultures were then harvested by centrifugation (4000× *g*, 10 min, 4 °C). The obtained bacterial pellets were resuspended in buffer R at room temperature to approximately the same initial OD_600_. In total, 100 µL of buffer R (controls) or 100 µL of CBD_TP84_28_His solution (0.145 mg/m) was added to 10 mL of the freshly prepared bacterial suspensions to the final concentration of 1.43 µg/mL and shaken at 55 °C. Moreover, 1 mL samples were taken at time intervals, and OD_600_ was measured.

- Turbidity reduction assay (Tecan microplate reader variant)

Overnight (16 h), a culture of *G. stearothermophilus* strain 10 was grown in LB medium at 55 °C. It was used to inoculate 30 mL of fresh LB medium, which was cultivated until the mid-exponential phase (OD_600_ = 0.6). The culture was harvested by centrifugation (4000× *g*, 10 min, 4 °C). The resulting bacterial pellet was resuspended in buffer R at room temperature to approximately the same optical density as the initial culture, according to the Tecan 96-well plate reader measurement. In total, 95 µL of the freshly prepared bacterial suspension was placed in the consecutive 14 wells. Then, 5 µg of buffer R (control) or 5 µL of 0.5 mg/mL CBD_TP84_28_His in buffer R (preincubated at different temperatures, as described in Section 2.2.4 was added to each well to a final concentration of 25 µg/mL. Measurements of OD_600_ were performed in 5 min intervals simultaneously for all the wells.

- Inhibition of biofilm formation by CBD_TP84_28_His

The ability of the fusion protein CBD_TP84_28_His to prevent or disrupt biofilm formation by *E. coli*, *S. aureus*, *P. aeruginosa*, *S. enteritidis*, *B. cereus* and *G. stearothermophilus* strain 10 was evaluated using a quantitative spectrophotometric microtiter plate assay as described before [11]. The selected bacterial culture was diluted with TSBg medium in the ratio 1:150 and left for 24 h at a temperature optimal for the given species (*E. coli*, *S. aureus*, *P. aeruginosa*, *S. enteritidis*, and *B. cereus—*at 37 °C and *G. stearothermophilus* strain 10 at 55 °C). Suspensions (4 mL each) were poured into consecutive wells of a 6-well flat-bottom polystyrene microplate. CBD_TP84_28_His was then added in amounts ranging from 0.05 to 50 µg (which corresponded to the final concentration of 0.0125 12.5 µg/mL) when efficacy against biofilm formation by *G. stearothermophilus* strain 10 was analysed. For other bacterial substrates, 2 µg (0.5 µg/mL) was used. The plates were incubated for 24 h at either 55 °C (*G. stearothermophilus* strain 10) or at 37 °C (*E. coli*, *S. aureus*, *P. aeruginosa*, *S. enteritidis*, *B. cereus*). After the initial incubation, the plate containing mesophilic bacteria was further incubated for 1 h at 55 °C to elevate the CBD_TP84_28_His to its optimal activity temperature. The plates were washed with 4 mL of 1 ×PBS buffer (137 mM NaCl; 10 mM Na_2_HPO_4_; 1.8 mM KH_2_PO_4_; 2.7 mM KCl, pH = 7.4) and stained with 0.1% crystal violet in 33% (*v*/*v*) acetic acid for 15 min, then washed twice with 4 mL of 1 × PBS. OD_600_ was measured with a Spectra Wallac microplate reader. TSBg and bacteria not treated with CBD_TP84_28_His were used as a negative control.

#### 4.3.2. Evaluation of the Thermostability of the CBD_TP84_28_His

To assess the thermostability of the CBD_TP84_28_His, 100 µL samples were incubated for 30 min in a thermocycler at selected temperatures (37.7 °C, 45.7 °C, 50.0 °C, 57.5 °C, 65.1 °C, 70 °C, 74.8 °C, 81.2 °C, 86.2 °C, 90.0 °C, 95.8 °C, and 100 °C for 30 min and in an autoclave at 121 °C for 20 min). Then, the TRA was performed as described in Section 4.3.1, and the data on lytic activity were collected for each of the preincubated samples. Additionally, nano-DSF was used to measure the unfolding temperature of the fusion CBD_TP84_28_His.

#### 4.3.3. Cellulose-Binding Properties of CBD_TP84_28_His

- Interaction with µC assay

We have devised methods for assessing the cellulose-binding properties of CBD_TP84_28_His in the format of it interaction with µC [42], patent application WIPO ST 10/C PL446913. A high-binding flat 96-well plate was coated with a suspension of µC (100 mg in 100 µL 1 × PBS) and incubated for 18 h at 4 °C. The plate was then centrifuged at 4000× *g* for 10 min at 4 °C. Serial dilutions of enzymes, starting at 1 µg per well, were applied to row A (TP84_28_His) and row B (CBD_TP84_28_His) in a volume of 100 µL and incubated at 4 °C for 18 h. The plate was then centrifuged and the supernatant extracted. The wells were washed 3 times with 1 × PBS by adding the solution and centrifuging the plate and incubated with a 100 µL blocking buffer per 5 min. Then, 100 µL of 1 × TBST (0.05 M Tris-HCl pH = 7.5, 0.15 M NaCl, 0.1% Tween 20) was added, and the plate was shaken for 5 min at room temperature. The wells were again vortexed and washed 3 times with TBST buffer by vortexing the plate. The complexes formed were resuspended in 100 µL of a 1:2000 solution of anti-His HRP-conjugated antibodies and incubated for 1 h at room temperature. They were centrifuged again, and the wells were washed 3 times with a TBST buffer. A DAB solution was then added to the washed wells to visualize the formed µC-CBD_TP84_28_His complexes by the HRP-DAB chemical reaction (a semi-quantitative method) (Figure 7).

- Cellulose paper-based immunoblotting assay

The cellulose-binding properties of CBD_TP84_28_His were also assessed using cellulose paper-based immunoblotting, a previously described method [43]. The procedure was run in parallel for CBD_TP84_28_His and the controls: recombinant TP84_28_His [19] and PBS. The solution with CBD_TP84_28_His was spotted onto cellulose paper and allowed to adsorb for 2 min at room temperature, which was followed by 10 min of incubation of the sample with 5 mL of blocking buffer (BSA solution). The sample was incubated for 1 h at 30 °C with anti-His HRP-conjugated antibodies, diluted at 1:2000 in 3 mL of 1 × TBST buffer. The sample was washed with 5 mL of 1 × TBST for 5 min. This step was repeated 3 times, and premixed DAB solution was spotted onto the paper. The appearance of dark spots indicated the successful detection of the His-tag (Figure 8).

### 4.4. Statistical Analysis

The statistical analysis of the data was performed using the Prism 9.0 software package (GraphPad Software, Inc., San Diego, CA, USA). Student’s *t*-test for dependent samples was used to assess how the parameters under study changed over time. Differences between the groups were analysed with a one-way ANOVA followed by Dunnett’s post hoc test or two-way ANOVA followed by Tukey’s post hoc test. The level of statistical significance was set at *p*-value < 0.05. The results were expressed as means ± SD of three independent experiments.

## 5. Conclusions

In this paper, we constructed and characterized a novel fusion protein, CBD_TP84_28_His, which exhibited the following properties:

(a) Enhanced thermostability: CBD_TP84_28_His exhibits significant thermostability, providing a marked advantage over the T4 lysozyme and other mesophilic endolysins. This makes it ideal for applications involving high heat exposure where mesophilic enzymes are ineffective.
(b) Targeted antimicrobial activity: CBD_TP84_28_His exhibits high lytic activity against thermophilic Gram-positive bacteria such as *Geobacillus* ICI, *B. stearothermophilus* and *T. aquaticus* and limited activity against mesophilic Gram-positive *Bacillus* species.
(c) The inhibition of biofilm formation: CBD_TP84_28_His effectively suppresses biofilm formation by pathogens such as *S. aureus* and *P. aeruginosa,* which pose a significant challenge in clinical settings. This underscores the potential of CBD_TP84_28_His in combating multi-drug-resistant infections.
(d) Compatibility with cellulose-based materials: Its fusion with CBD allows CBD_TP84_28_His to anchor onto cellulose-based surfaces, providing long-lasting, localized antimicrobial activity. This property makes it particularly suitable for use in wound dressings, food packaging, and cellulose-based medical devices (patent application WIPO ST 10/C PL446913; WIPO ST 10/C PL449273) (Figure 7 and Figure 8).
(e) Potential for broad applications: beyond healthcare, CBD_TP84_28_His shows promise in the food industry, offering a safer alternative to conventional preservatives (patent application WIPO ST 10/C PL449273).

## 6. Patents

Related patent applications of our research group include the following: (i) the µC synthesis method—WIPO ST 10/C PL446913; (ii) fusion recombinant protein CBD_TP84_28_His; the method of obtaining it and its application was published in the following work: WIPO ST 10/C PL449273.

## Figures and Tables

**Table 1:** Inhibition of biofilm formation by *E. coli*, *S. aureus*, *P. aeruginosa*, *S. enteritidis*, *B. cereus. The table* shows the relative OD_600_ value for a bacteria biofilm control process compared to a control reaction devoid of CBD_TP84_28_His. Biofilm of each bacterium was treated with 2 µg of CBD_TP84_28_His at 37 °C for 24 h and at 55 °C for 1 h. Results are presented as means ± SD of three independent experiments. Statistically significant differences were determined by Student’s t-test and marked as (*)—*p*-value < 0.05, (**)—*p*-value < 0.01, (***)—*p*-value < 0.001.

Bacteria	Control [OD_600_]	Treatment [OD_600_]	Reduction [%]	*p*-Value
*E. coli* (DSM 1103)	5.5 ± 0.10	3.15 ± 0.15	39	0.0675
*S. aureus* (ATCC 25923)	1.3 ± 0.30	0.225 ± 0.075	81 *	0.0143
*P. aeruginosa* (ATCC 17503)	12.45 ± 2.45	6.35 ± 2.25	42 ***	0.0004
*S. enteritidis* (ATCC 25928)	0.135 ± 0.005	0.08 ± 0.01	34 **	0.0034
*B. cereus* (DSM 31)	9.05 ± 2.85	4.9 ± 3.10	33 **	0.0012

## Data Availability

All data generated or analysed during this study are included in this published article. The datasets used and/or analysed during the current study are available from the corresponding author on reasonable request.

## Supplementary Materials

The following supporting information can be downloaded at: https://www.mdpi.com/article/10.3390/ijms252313111/s1.
