# Supplementary material for: New-Generation Antibacterial Agent—Cellulose-Binding Thermostable TP84_Endolysin
Source: Int J Mol Sci. 2024 Dec 6;25(23):13111. doi: 10.3390/ijms252313111 (PMC11642820; doi:10.3390/ijms252313111)
Supplement: Supplementary file 1 [file ijms-25-13111-s001.zip › ijms-3309738-supplementary.pdf]

SUPPORTING INFORMATION

International Journal of Molecular Sciences

New-Generation Antibacterial Agent—Cellulose-Binding Thermostable TP84_Endolysin

Małgorzata Ponikowska [1,2,†], Joanna Żebrowska [1,*,†] and Piotr M. Skowron [1,*]

[1] Department of Molecular Biotechnology, Faculty of Chemistry, University of Gdansk, 80-309 Gdansk, Poland; malgorzata.ponikowska@gumed.edu.pl

[2] Department of Biology and Medical Genetics, Faculty of Medicine, Medical University of Gdansk,
80-211 Gdansk, Poland

* Correspondence: joanna.zebrowska@ug.edu.pl (J.Ż.); piotr.skowron@ug.edu.pl (P.M.S.); Tel.: +48-585-235241 (J.Ż.); +48-585-235242 (P.M.S.)

† These authors contributed equally to this work.

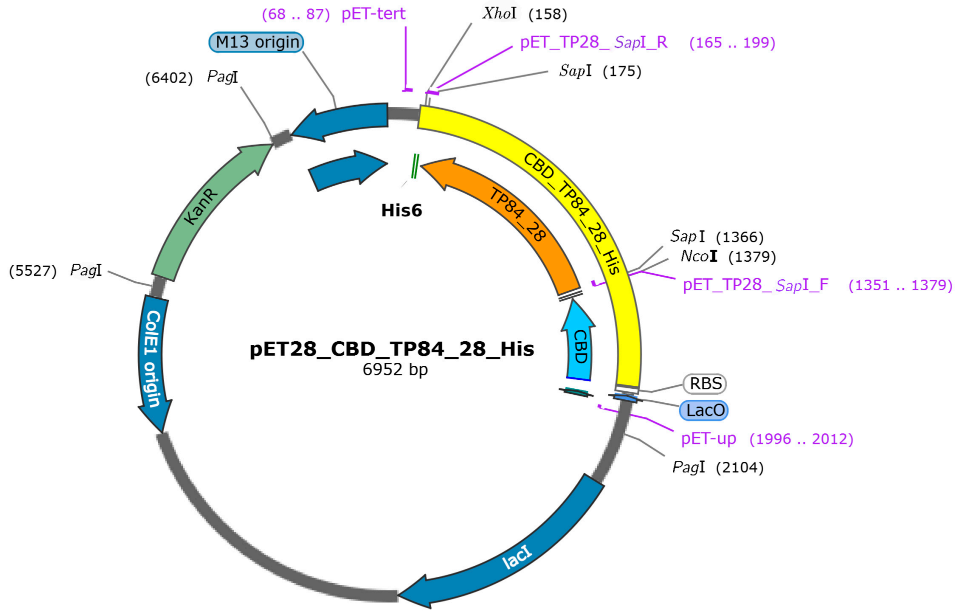

Figure S1. Plasmid pET28_CBD_TP84_28_His.TP84_28 – gene coding for the endolysin TP84_28; CBD – gene coding for cellulose binding domain; CBD_TP84_28 – fusion gene which was formed after cloning TP84_28 into the plasmid pET28_CBD_His; LacO – operator LacO; RBS – ribosome binding domain; pET-up, pET-tert – attachment sites for pET_up and pET_tert primers correspondingly; pET _TP28_*Sap*I_R, pET_TP28_*Sap*I_F – attachment sites for pET_TP28_*Sap*I_R and pET_TP28_*Sap*I_F primers correspondingly; *Pag*I, *Xho*I, *Sap*I, *Nco*I – restriction site for *Xho*I, *Sap*I, *Nco*I; KanR – gene coding for k anamycin resistance; ColE1 origin – origin of replication , lacI – *lac* repressor-coding gene, M13 origin – replication origin for ssDNA M13 bacteriophage , His6 – sequence encoding histidine tag.

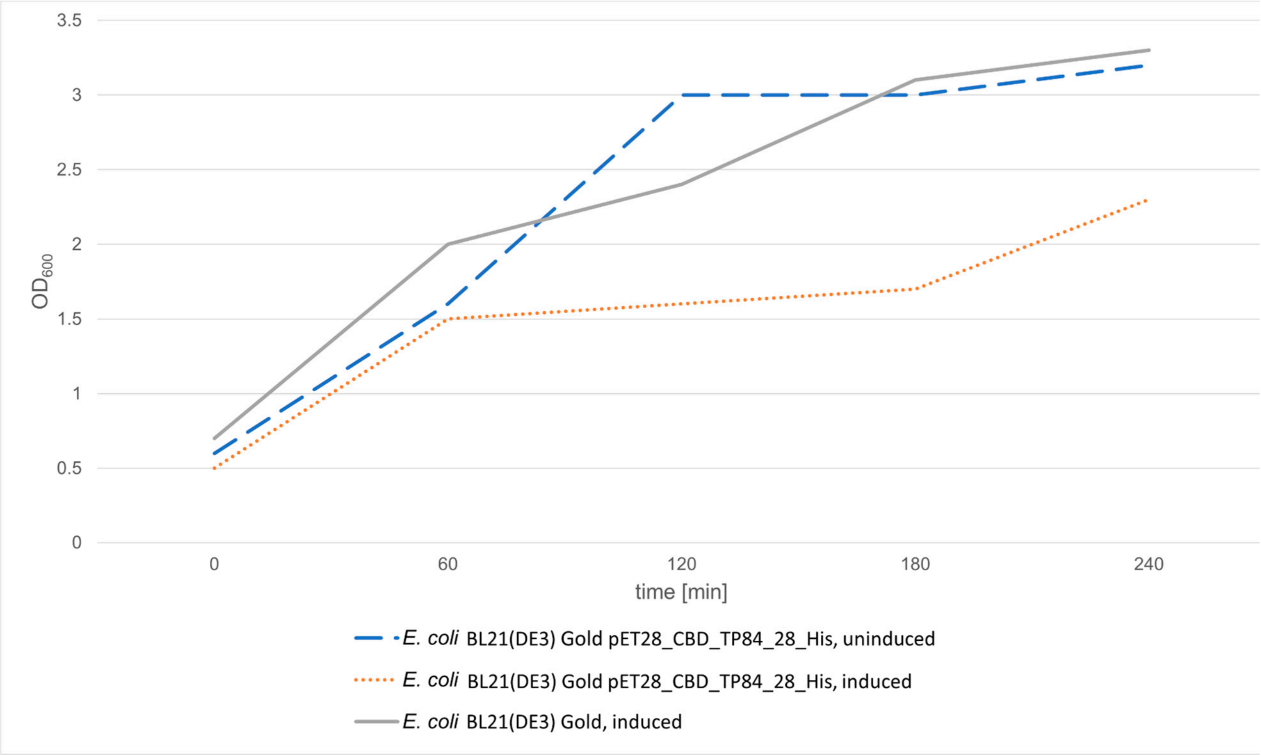

Figure S2. **Growth curve of *E. coli* BL21(DE3) Gold with recombinant plasmid pET28delSapI_CBD_TP84_28_His after induction of fusion gene *cbd_tp84_28* expression and controls** *E.coli* BL21(DE3) Gold pETdelSapI_CBD_TP84_His**,** uninduced **-** expression strain *E.coli* BL21(DE3) with recombinant plasmid pET28delSapI_TP84_28_His not induced with IPTG ; *E.coli* BL21(DE3) Gold pETdelSapI_CBD_TP84_28_His**,** induced - expression strain *E. coli* BL21(DE3) with recombinant plasmid pET28delSapI_TP84_28_His induced with IPTG; *E. coli* BL21(DE3) Gold**,** induced **-** expression strain *E. coli* BL21(DE3) without recombinant plasmid induced with IPTG.

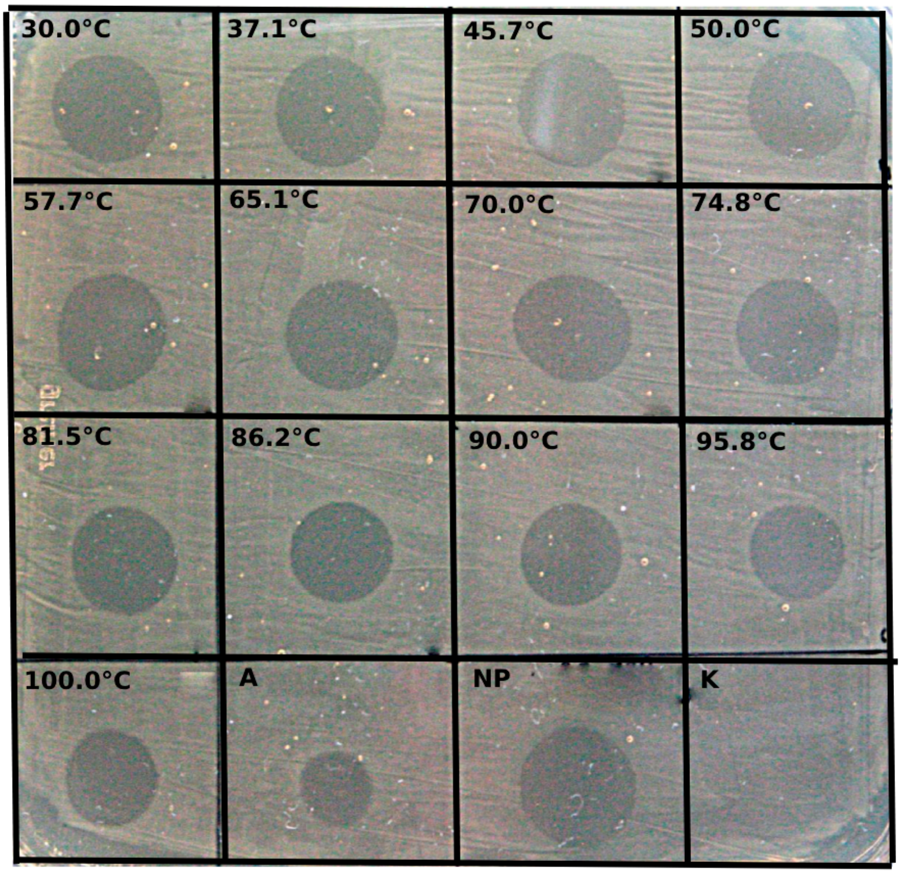

Figure S3. Recombinant fusion endolysin CBD_TP84_28_His thermostability determination – spot assay.

CBD_TP84_28_His preparations preincubated at temperatures of 30-100°C, autoclaved (A), not preincubated (NP), and buffer R (K) were applied to the agar plate with freshly spread *G. stearothermophilus* strain 10 cells, followed by incubation of the plate at 55°C for 10 h. Transparent circles on the bacterial lawn reveal the lytic effect of the recombinant enzyme.
